# Supplementary material for: Sustainability-inspired cell design for a fully recyclable sodium ion battery
Source: Nat Commun. 2019 Apr 29;10:1965. doi: 10.1038/s41467-019-09933-0 (PMC6488666; doi:10.1038/s41467-019-09933-0)
Supplement: Supplementary file 1 — Supplementary Information [file 41467_2019_9933_MOESM1_ESM.pdf]

Supporting Information

Sustainability Inspired Cell Design for A Fully Recyclable Sodium Ion Battery

Liu et al.

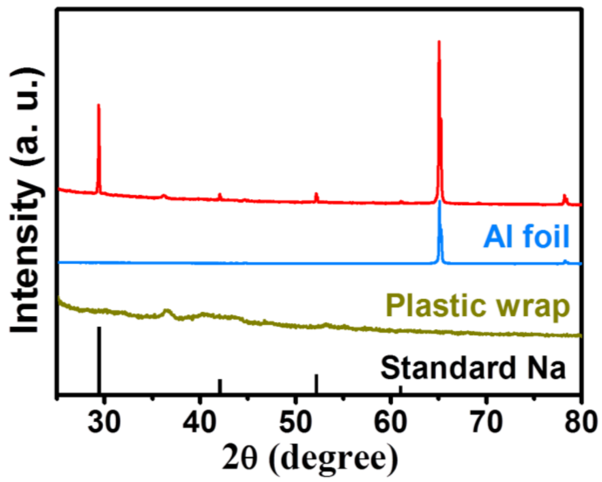

Supplementary Figure 1 | Ex-situ XRD spectra of deposited metallic Na on the surface of Al foil after discharging. Three separate phases exist in the sample, i.e. metallic Na (JCPDS No. 22-0948), Al foil, and plastic wrap film. Plastic wrap film was used to prevent the oxidation of metallic Na in the air during testing.

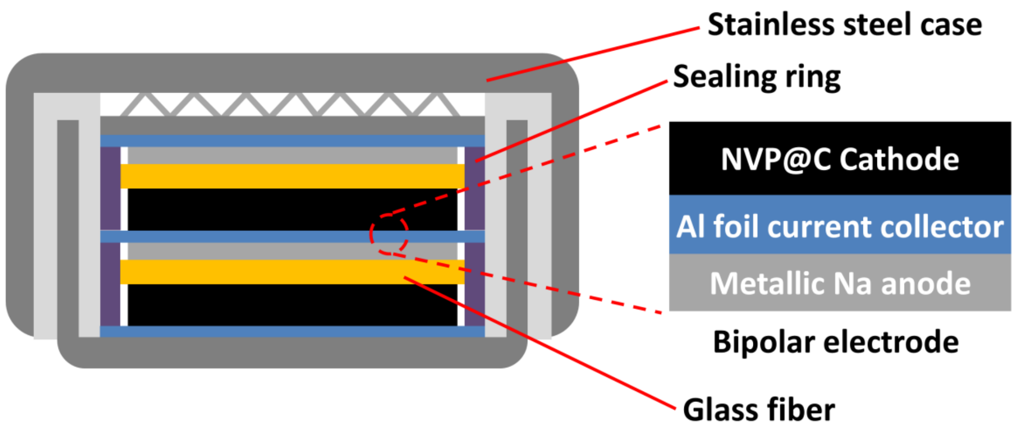

Supplementary Figure 2 | A simple schematic of a bipolar NIB cell with two units. Al foil is an shared current collector for both the cathode and anode. The electrolyte is sealed in each cell by sealing ring and current collector.

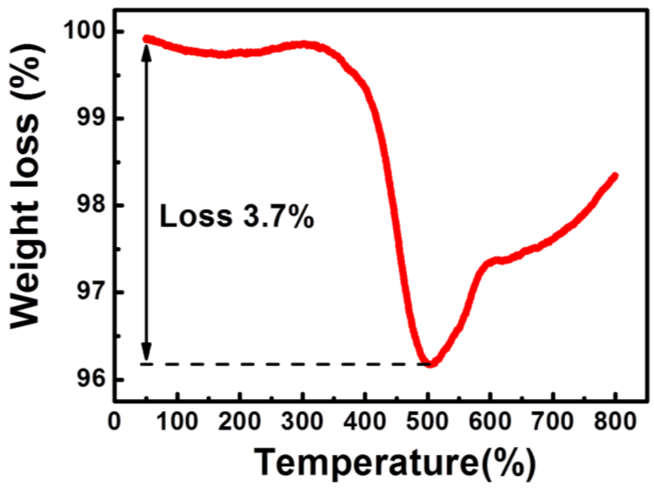

Supplementary Figure 3 | TGA curves of the NVP@C for confirming carbon content in the as-prepared sample. The increase in the weight after 500 ºC is attributed to the oxidation of elemental V burned in the air.

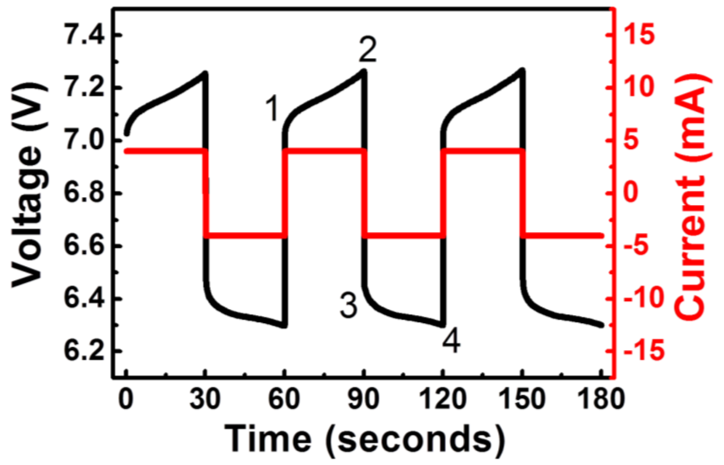

Supplementary Figure 4 | Current and voltage curves of a two-unit bipolar NIB cell for high charge/discharge tests at a 20 C rate. Numbers 1, 2, 3, and 4 denote the initial voltage of fast charging, end voltage of fast charging, initial voltage of fast discharging, and end voltage of fast discharging, respectively.

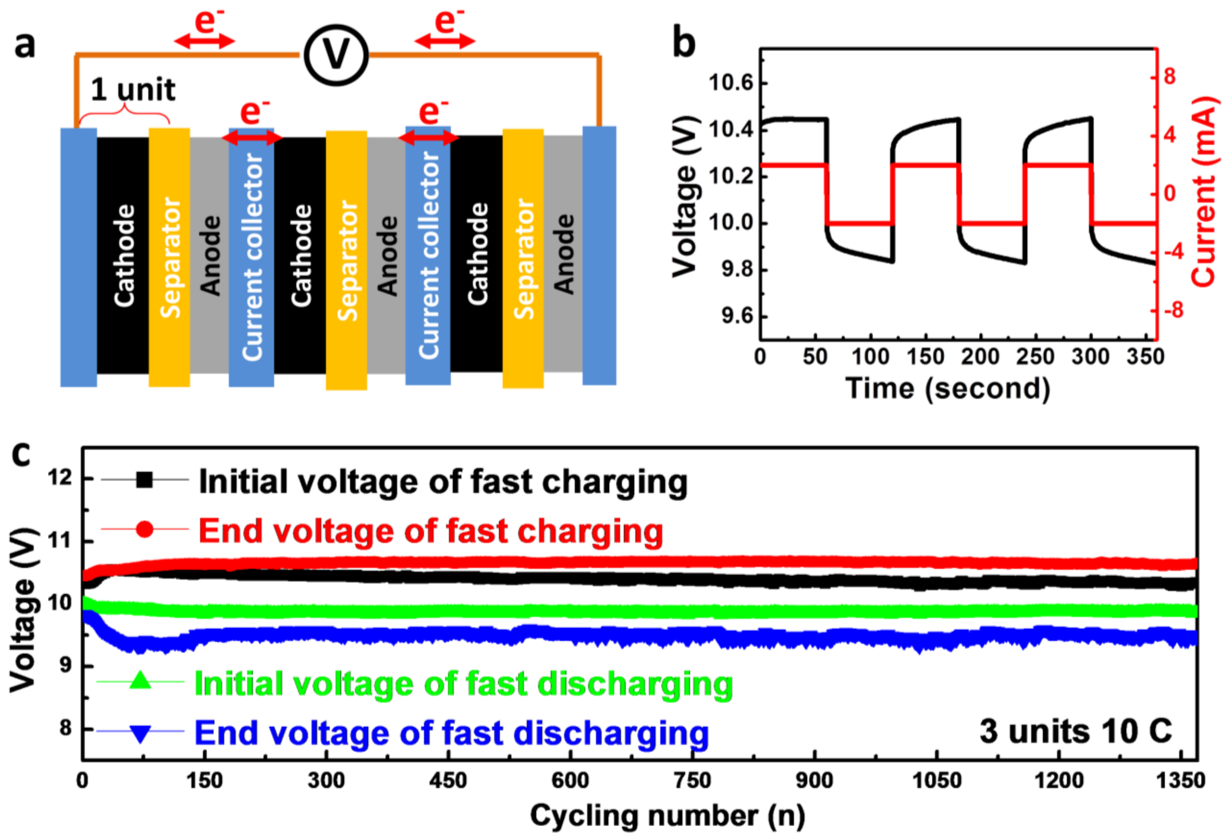

Supplementary Figure 5 | Design and High charge/discharge tests of a three-unit bipolar NIB cell at 10 C rate for 60 s. (a) A schematic of the electrode structure in a battery pack. (b) Current and voltage curves in the charge and discharge process. (c) The voltages of long cycles at the beginning and end of each step.

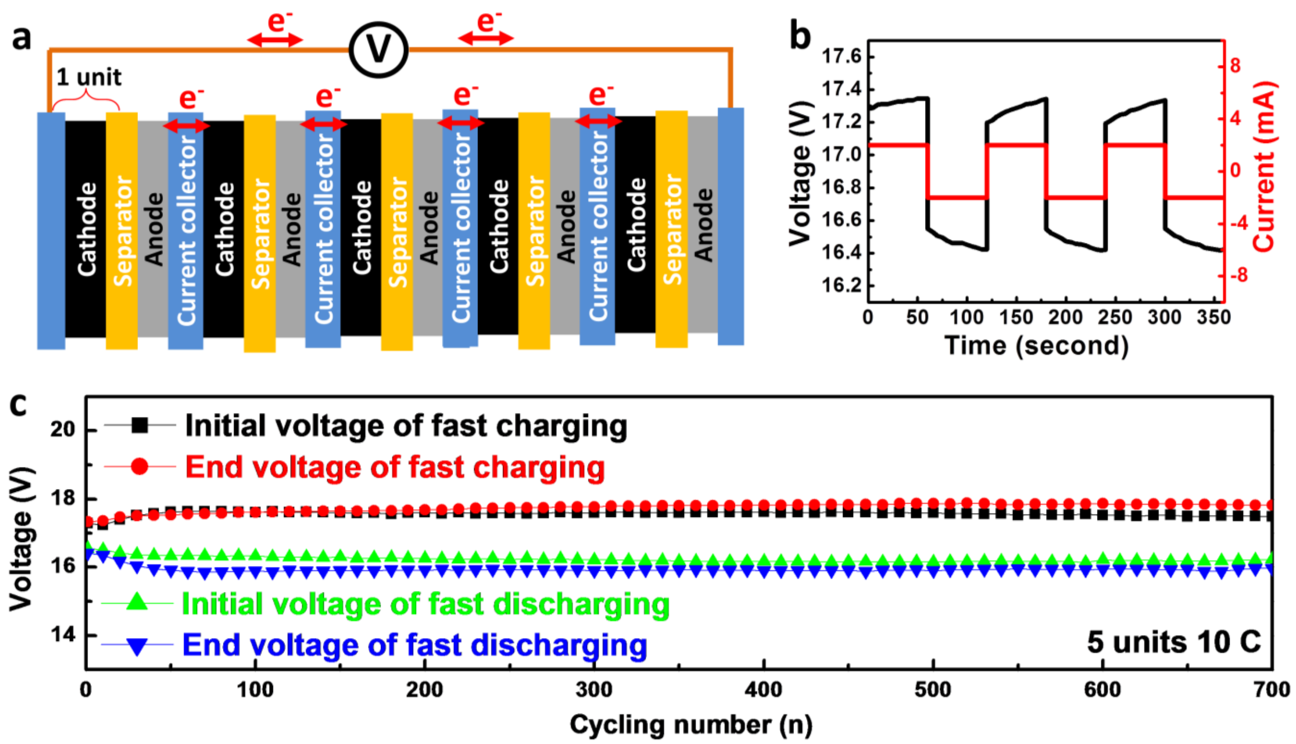

Supplementary Figure 6 | Design and high charge/discharge tests of a five-unit bipolar NIB cell at a 10 C rate for 60 s. (a) The simple schematic of the cell structure. (b) The current-time and voltage-time curves in galvanic charge and discharge processes. (c) The voltages of long cycles at the beginning and end of each step.

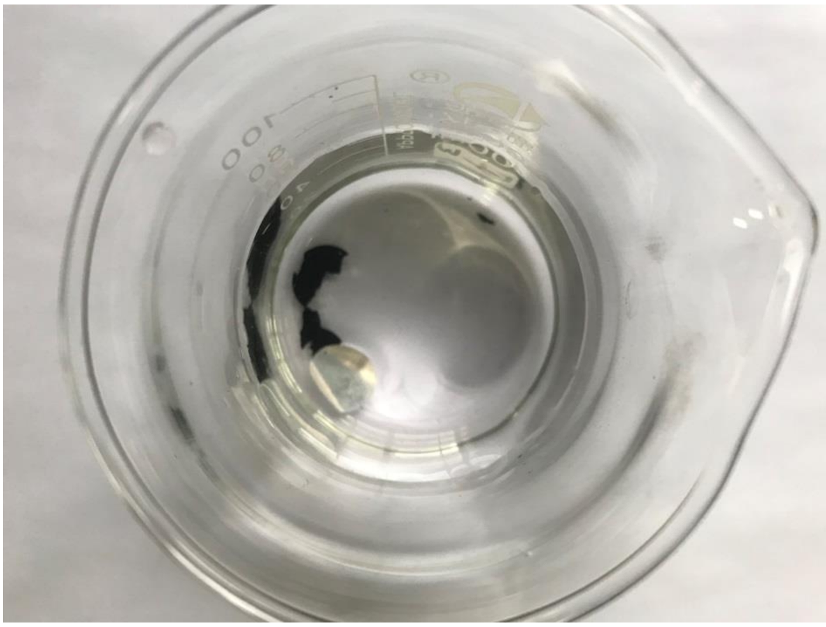

Supplementary Figure 7 | The separation of electrode slurry and Al foil in recycling process.

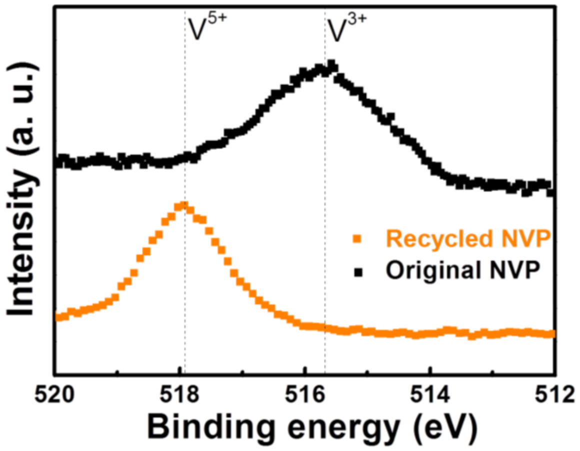

Supplementary Figure 8 | Ex-situ XPS studies of original NVP@C and recycled NVP powder.

XPS was used to examine the oxidation states of elemental V in the samples. The original NVP-based material displays a peak located at the binding energy of 515.6 eV, corresponding to V^{3+}. After heat treatment in air, the binding energy of V^{3+} increases to 517.9 eV, indicating the oxidation of elemental V in recycled NVP.[1] This result is consistent with the TGA analysis in Figure S3.

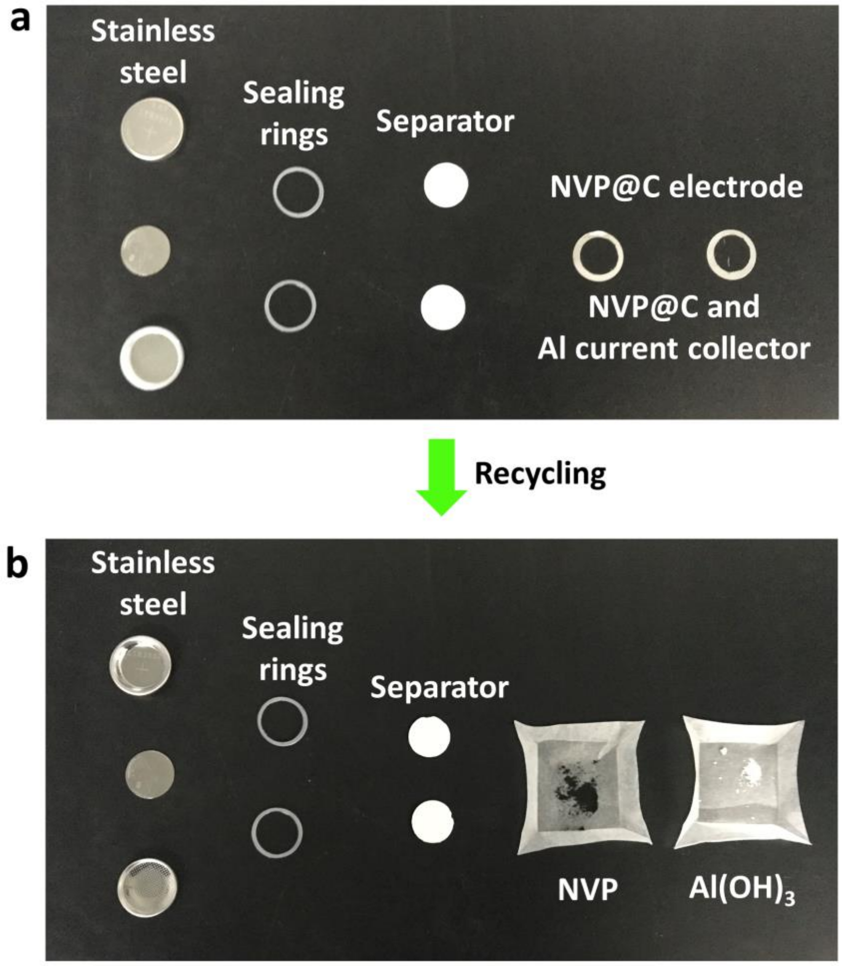

Supplementary Figure 9 | Optical images of the original and recycled components of an asymmetric bipolar NIB cell with NVP@C cathode and metallic Na anode. (a) Original components except metallic Na anode and organic electrolyte. (b) Recycled components containing $Al(OH)_3$ to recycle the Al element.

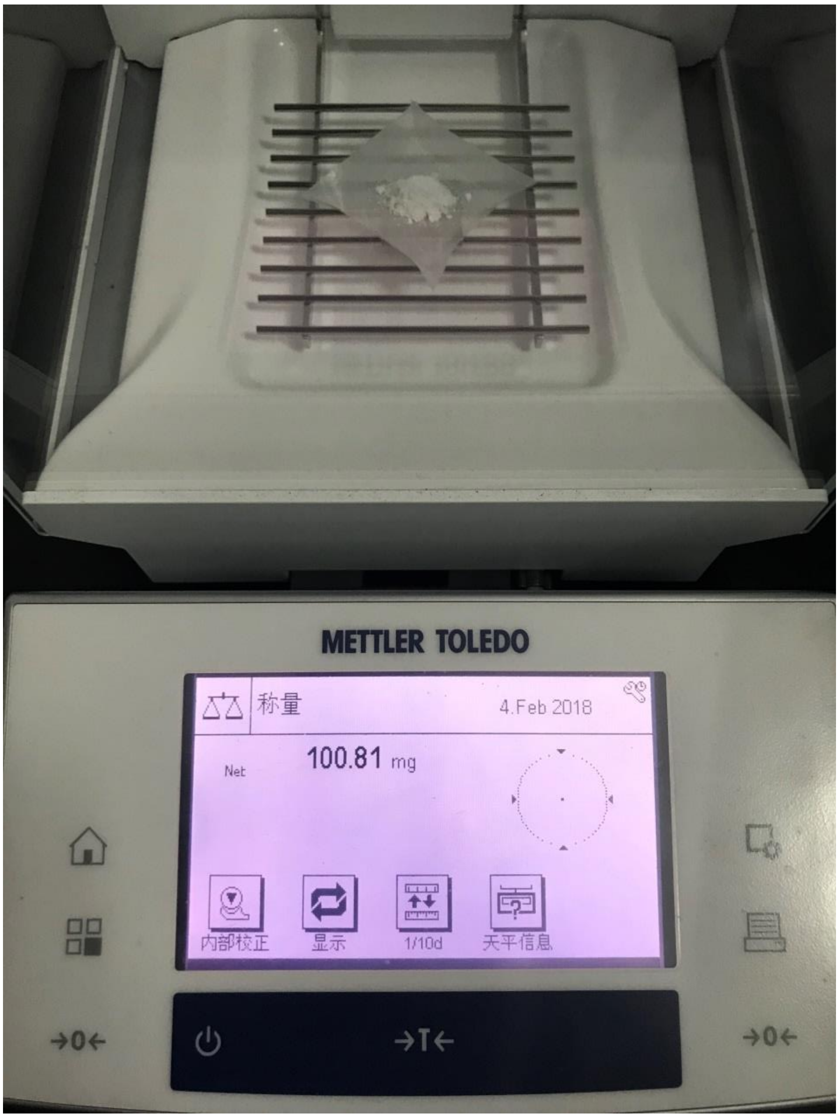

Supplementary Figure 10 | Weight of the recycled $Al(OH)_3$ of a typical two-unit cell after filtration and drying. The recycling rate of elemental Al is calculated by the weight ratio of Al content in recycled $Al(OH)_3$ and used Al foil.

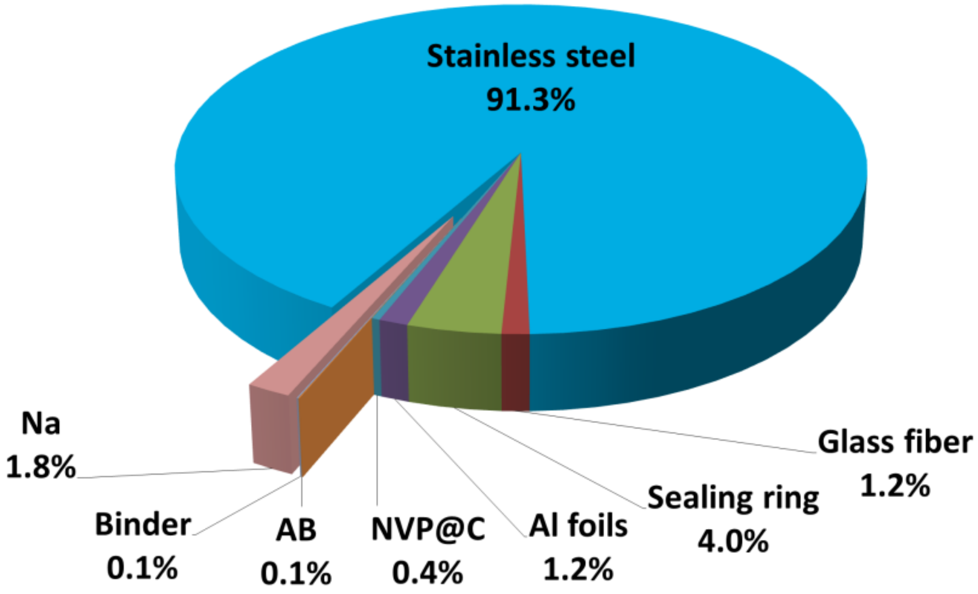

Supplementary Figure 11 | A schematic diagram of weight breakdown of a bipolar NIB with NVP@C cathode and metallic Na anode. The areas separated from the disc are not recycled. With the exception of elemental Al with recycling rate of *ca*. 99.1%,[3] main components are recycled at almost 100%.

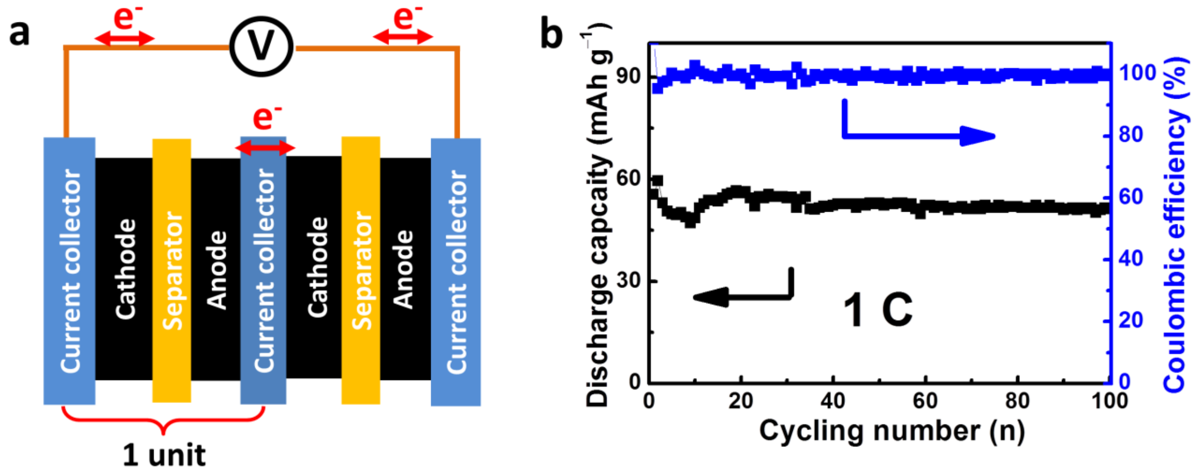

Supplementary Figure 12 | Design and electrochemical behavior of a two-unit bipolar NIB cell using identical materials for cathode and anode. (a) Electrode structure and (b) electrochemical performance of a symmetric two-unit bipolar NIB cell. The cycling of the symmetric cell at 10 C in the voltage range from 2 to 4.4 V.

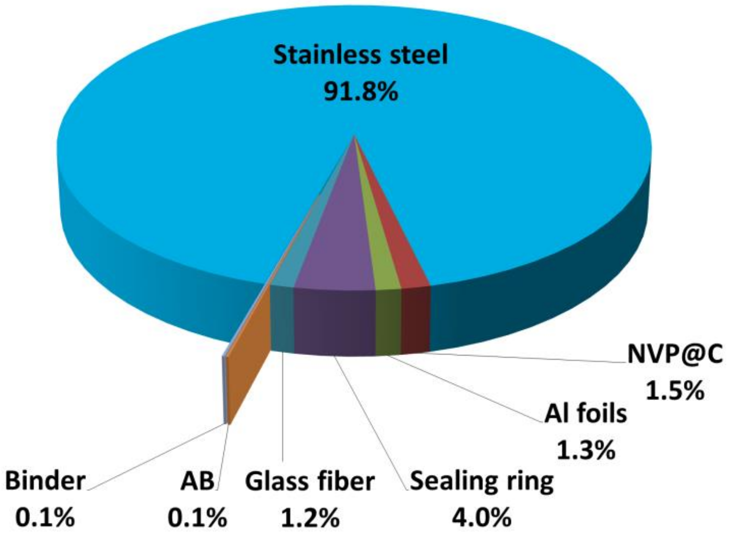

Supplementary Figure 13 | A schematic diagram of weight breakdown of a bipolar NIB using NVP@C as the cathode and anode. The area separated from the disc is not recycled. With the exception of elemental Al with recycling rate of *ca.* 99.1%,[3] the main components are recycled at almost 100%.

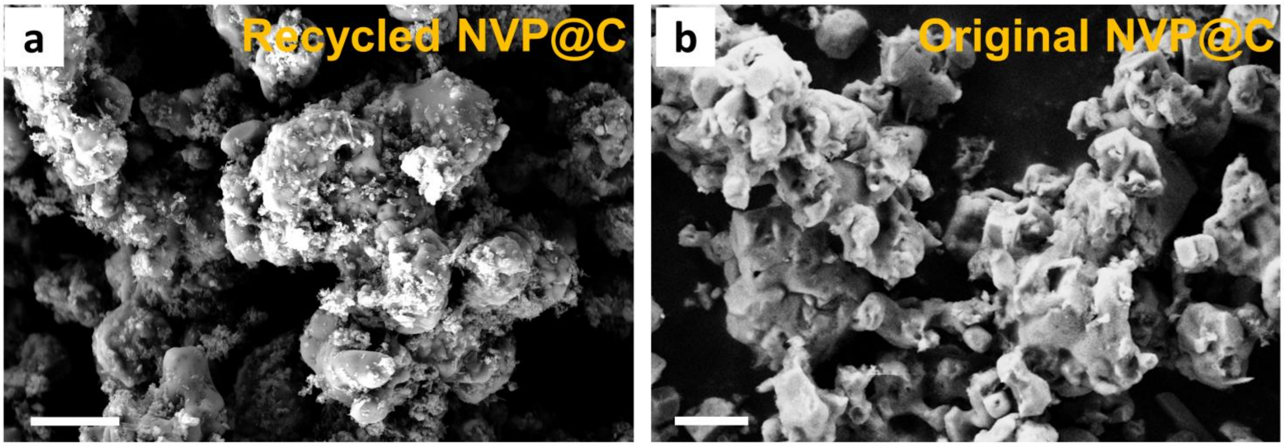

Supplementary Figure 14 | The morphology of the NVP@C. (a) Recycled NVP@C and (b) original NVP@C. The scale bar in both images is 1 μm.

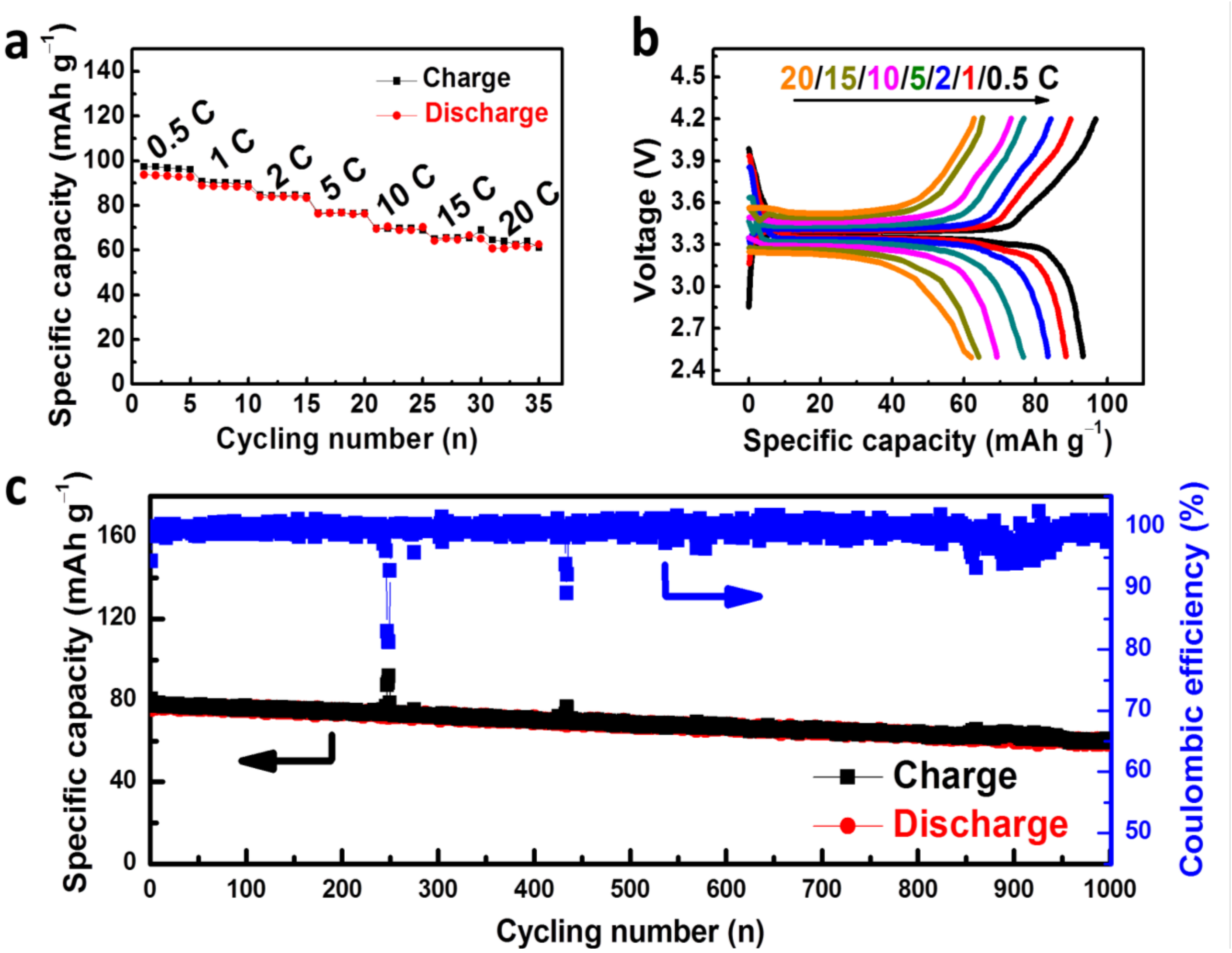

Supplementary Figure 15 | Electrochemical behavior of the recycled NVP@C composite. (a) Rate performance from 0.5 to 20 C. (b) Corresponding charge and discharge curves at different rates. (c) Long cycle life with 1000 cycles at the rate of 10 C.

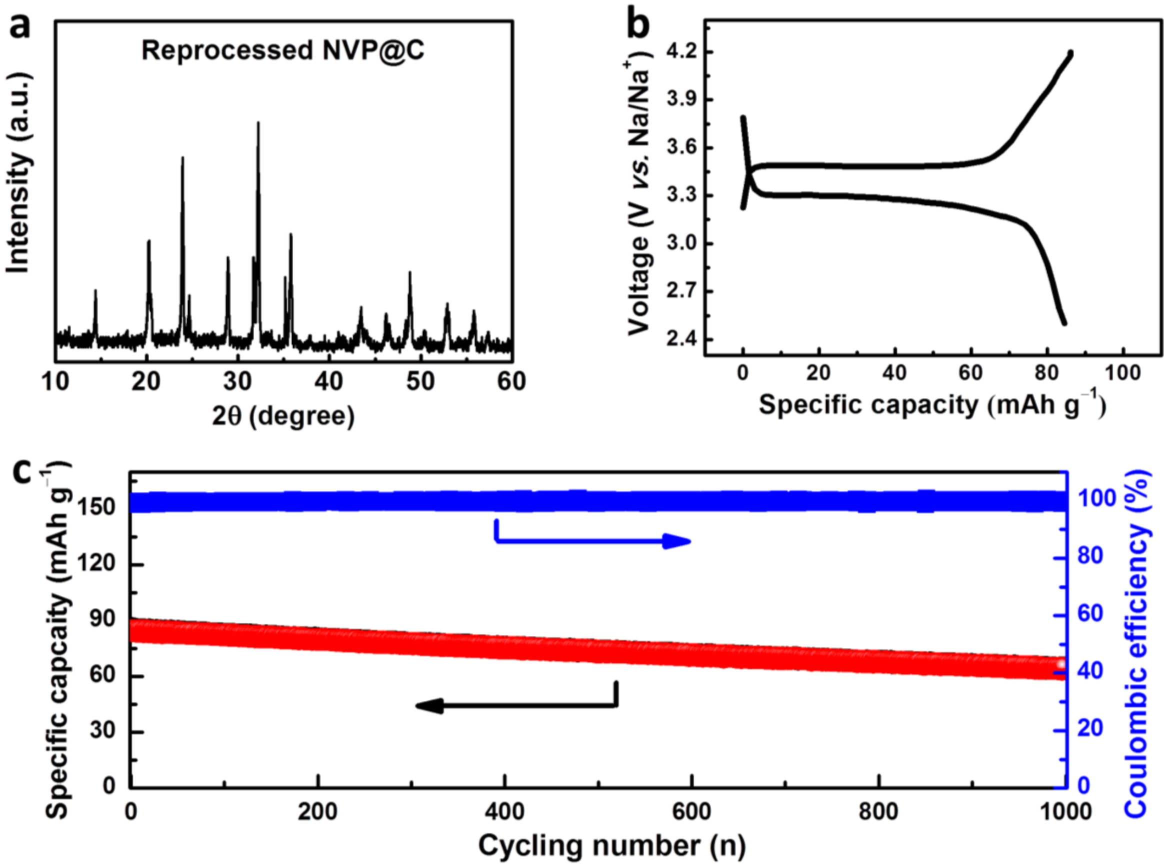

Supplementary Figure 16 | The NVP@C composite at secondary recycling. (a) XRD spectrum; (b) The charge and discharge curves at 5 C; (c) Cycle performance with 1000 cycles at the rate of 5 C.

Supplementary Table 1 | Weight breakdown of a typical bipolar NIB with metallic Na anode as shown in Supplementary Fig. 11.

Item		Component	Initial Weight (g)	Result	Recycled Weight (g)	Recycling Rate (%)[a]
**Accessories**		Stainless steel cases	2.4876	recycle	2.4876	100
		Sealing rings	0.1091	recycle	0.1091	100
**Separator**		Glass fiber	0.0322	recycle	0.0322	100
**Electrolyte**[b]		Organic	-	-	-	-
**Unit**	**Cathode**	NVP[c]	0.0096	recycle	0.0096	100
		Acetylene black	0.0012	burn	-	-
		Polymer binder	0.0012	burn	-	-
	**Anode**	Metallic Na	0.0498	form NaCl	-	-
	**Current Collector**	Al foil	0.0339	recycle	0.0336	99.1[d]
		**Total**	**2.7246**		**2.6721**	**98.1**

[a]Recycling rate is defined as the weight ratio of acceptable recycling products and considered battery scrap mass.[7]

[b]Here we do not consider the recovery of organic electrolyte, see work by Georgi-Maschler et al.[7]

[c]NVP is coated with 3.7 wt.% carbon layer as shown in Supplementary Figure 3, which is used to enhance the electronic conductivity of the NVP-based composite.

[d]This cycling rate is based on the calculation of elemental Al in the form of $Al(OH)_3$ in an acid-base neutralization reaction in our work (Supplementary Figure 7), which can be enhanced to ~100% by tuning the pH value to decrease the concentration of Al^{3+} ions down to the theoretical value of 10^{-5} in the solution.

Supplementary Table 2 | Recovery comparisons between traditional methods and this work.

Items \ Method	Hydro-metallurgical recovery	Pyro-metallurgical recovery	Direct recovery	This work
**Recycling cost**	high	low	high	low
**Environmental effect**	serious	moderate	moderate	hardly
**Energy consumption**	low	high	low	low
**Reprocessing cost**	high	high	low	low
**Possibility of sustainability**	low	low	low	low

Supplementary Table 3 | Weight breakdown of a typical bipolar NIB with an NVP@C anode as shown in Supplementary Figure 13.

Item		Component	Initial Weight (g)	Result	Recycled Weight (g)	Recycling Rate (%)[a]
**Accessories**		Stainless steel cases	2.4872	recycle	2.4872	100
		Sealing rings	0.1091	recycle	0.1091	100
**Separator**		Glass fiber	0.0322	recycle	0.0322	100
**Electrolyte[b]**		Organic	-	-	-	-
**Unit**	**Cathode and Anode[d]**	NVP[c]	0.0297	recycle	0.0297	100
		Polymer binder	0.0037	burn	-	-
		Acetylene black	0.0037	burn	-	-
	**Current Collector**	Al foil	0.0342	recycle	0.0339	99.1[e]
		**Total**	**2.6998**		**2.6921**	**99.7**

[a]Recycling rate is defined as the weight ratio of acceptable recycling products and considered battery scrap mass.[7]

[b]Here we do not consider the recovery of organic electrolyte, see work by Georgi-Maschler et al.[7]

[c]NVP is coated with 3.7 wt.% carbon layer as shown in Supplementary Figure 3, which is used to enhance the electronic conductivity of the NVP-based composite.

[d]The weight ratio of the cathode active material and anode active material is 1:2.

[e]This cycling rate is based on the calculation of elemental Al in the form of $Al(OH)_3$ in an acid-base neutralization reaction in our work, which can be enhanced to ~100% by tuning the pH value to decrease the concentration of Al^{3+} ions down to the theoretical value of 10^{-5} in the solution.

Supplementary Note 1 | The calculation of the recycling rate of Al source according to the results from Supplementary Figure 10.

Recycled mass of the Al $= \frac{26}{78} \times 0.10081\ g = 0.0336g$

Recycling rate of the Al source $= \frac{0.0336\ g}{0.0339g} \times 100\% = 99.1\%$

Supplementary Note 2 | Principle and electrochemical behavior of two-unit bipolar NIB cell using identical materials for cathode and anode

The symmetric two-unit bipolar NIB cell is assembled by two symmetric units. In each symmetric unit, the NVP@C is used as both the cathode and the anode. Like other symmetric system[2-6], the chemical reactions in the full units of NVP@C can be described as follows:

During charging,

Cathode: $Na_3V_2^{3+}(PO_4)_3 \rightarrow Na_{3-x}V_{2-x}^{3+}V_x^{4+}(PO_4)_3 + xNa + xe^- \rightarrow NaV_2^{5+}(PO_4)_3$

Anode: $Na_3V_2^{3+}(PO_4)_3 + xNa^+ + xe^- \rightarrow Na_{3+x}V_{2-x}^{3+}V_x^{2+}(PO_4)_3 \rightarrow Na_5V_2^{2+}(PO_4)_3$

Overall: $Na_3V_2^{3+}(PO_4)_3 \rightarrow Na_5V_2^{2+}(PO_4)_3 + NaV_2^{5+}(PO_4)_3$

During discharging,

Cathode: $NaV_2^{5+}(PO_4)_3 \rightarrow Na_{3-x}V_{2-x}^{3+}V_x^{4+}(PO_4)_3 + xNa + xe^- \rightarrow Na_3V_2^{3+}(PO_4)_3$

Anode: $Na_5V_2^{2+}(PO_4)_3 \rightarrow Na_{3+x}V_{2-x}^{3+}V_x^{2+}(PO_4)_3 \rightarrow Na_3V_2^{3+}(PO_4)_3 + xNa^+ + xe^-$

Overall: $Na_5V_2^{2+}(PO_4)_3 + NaV_2^{5+}(PO_4)_3 \rightarrow Na_3V_2^{3+}(PO_4)_3$

Given that both cathode and anode use the initial active material of NVP@C, one symmetric unit exhibits the open-circuit voltage near 0 V. During the charging process, the cathode undergoes reversible electrochemical reactions of $Na_3V_2(PO_4)_3/NaV_2(PO_4)_3$, corresponding to the $V^{3+/}V^{4+}$ redox couple. The anode also performs the reversible electrochemical reaction of $Na_3V_2(PO_4)_3/Na_5V_2(PO_4)_3$, corresponding to the $V^{3+/}V^{2+}$ redox couple. Due to the 3.40 and 1.65 V corresponding to $V^{3+/}V^{4+}$ and $V^{3+/}V^{2+}$ redox couples, respectively, the output voltage of one symmetric NVP@C unit is *ca.* 1.70 V. Therefore, the symmetric two-unit bipolar NIB cell can deliver an output voltage of 3.40 V.

Supplementary Video 1 | Operation of the full recycling for a two-unit bipolar NIB with NVP@C cathode and metallic Na anode.

Supplementary Video 2 | Operation of the full recycling for a two-unit bipolar NIB with NVP@C as cathode and anode.

Supplementary References

1 Saravanan, K., Mason, C. W., Rudola, A., Wong, K. H. & Balaya, P. The first report on excellent cycling stability and superior rate capability of $Na_3V_2(PO_4)_3$ for sodium ion batteries. *Adv. Energy Mater.* **3**, 444−450 (2013).

2 Duan, W. *et al.* $Na_3V_2(PO_4)_3$@C core–shell nanocomposites for rechargeable sodium-ion batteries. *J. Mater. Chem. A* **2**, 8668−8675 (2014).

3 Li, S. *et al.* Effect of Carbon Matrix Dimensions on the electrochemical properties of $Na_3V_2(PO_4)_3$ nanograins for high-performance symmetric sodium-Ion batteries. *Adv. Mater.* **26**, 3545−3553 (2014).

4 Zhu, C., Kopold, P., van Aken, P. A., Maier, J. & Yu, Y. High power–high energy sodium battery based on threefold interpenetrating network. *Adv. Mater.* **28**, 2409−2416 (2016).

5 Jian, Z. *et al.* Carbon coated $Na_3V_2(PO_4)_3$ as novel electrode material for sodium ion batteries. *Electrochem. Commun.* **14**, 86−89 (2012).

6 Noguchi, Y., Kobayashi, E., Plashnitsa, L. S., Okada, S. & Yamaki, J.-i. Fabrication and performances of all solid-state symmetric sodium battery based on NASICON-related compounds. *Electrochim. Acta* **101**, 59−65 (2013).

7 Georgi-Maschler, T., Friedrich, B., Weyhe, R., Heegn, H. & Rutz, M. Development of a recycling process for Li-ion batteries. *J. Power Sources* **207**, 173−182 (2012).

8 Gratz, E., Sa, Q., Apelian, D. & Wang, Y. A closed loop process for recycling spent lithium ion batteries. *J. Power Sources* **262**, 255−262 (2014).

9 Xiao, J., Li, J. & Xu, Z. Recycling metals from lithium ion battery by mechanical separation and vacuum metallurgy. *J. Hazard. Mater.* **338**, 124−131 (2017).

10 Sabisch, J. E. C., Anapolsky, A., Liu, G. & Minor, A. M. Evaluation of using pre-lithiated graphite from recycled Li-ion batteries for new LiB anodes. *Resour. Conserv. Recy.* **129**, 129−134 (2018).
