# Supplementary material for: Sustainability-inspired cell design for a fully recyclable sodium ion battery
Source: Nat Commun. 2019 Apr 29;10:1965. doi: 10.1038/s41467-019-09933-0 (PMC6488666; doi:10.1038/s41467-019-09933-0)
Supplement: Supplementary file 3 — Description of Additional Supplementary Files [file 41467_2019_9933_MOESM3_ESM.pdf]

Description of Additional Supplementary Files

Supplementary Movie 1: Detailed operations and phenomena in the recycling process of a two-unit NIB cell.

Supplementary Movie 2: The recovery for a symmetric NIB cell.
